# T106. IMPROVING PSYCHIATRIC DIAGNOSIS BY ADDING MOTOR FUNCTION NEXT TO MENTAL HEALTH FUNCTION: A NETWORK APPROACH

**DOI:** 10.1093/schbul/sby016.382

**Published:** 2018-04-01

**Authors:** Galoeh Adrian Noviar, Didi Rhebergen, P Roberto Bakker

**Affiliations:** 1 Vrije Universiteit Amsterdam; 2 Maastricht University

## Abstract

**Background:**

Currently, the predictive value of psychiatric diagnosis is inadequate compared to other medical fields. It has been suggested that the use of a network model might aid in acquiring new insights into the underlying connections between symptoms (Bringmann et al., 2013). In addition, previous research (Bakker et al., 2012) has revealed associations between dysregulated mental- and motor function. As such, the network graphs might be enhanced by adding non-mental factors.

**Methods:**

Baseline data from a 4-year prospective naturalistic study (Bakker et al., 2012) was used to obtain data about 207 psychiatric long-stay patients. (i) Drug-induced movement disorders: tardive dystonia (TD), akathisia, parkinsonism, and dyskinesia. (ii) ratings of the clinical global impression-schizophrenia (CGI) scale, and (iii) age and total defined daily dose to account for potential confounders. Statistical programming environment R (Epskamsp, Cramer, Waldorp & Borsboom, 2012) was used to visualise several psychopathology-severity networks.

**Results:**

Interpretation of the graphs is based on the “centrality” of the symptoms. Centrality indicates the influence of a symptom on the network. Parkinsonism scored a low centrality score in graphs depicting high psychopathology in contrast with the other levels. Dyskinesia scored a low centrality score in medium psychopathology contrary to the other levels. The network graphs show a consistent positive correlation between age and parkinsonism (0.25, 0.53, and 0.19 for low, medium, and high psychopathology, respectively.), and a negative correlation between age and akathisia (-0.32, -0.47, and -0.21, respectively). High severities of psychopathology negatively correlated with parkinsonism (-0.16) and positively correlated with high levels of TD (0.33).

**Discussion:**

The usage of a network model including motor factors has provided useful information to take into consideration when examining psychopathology of a patient. TD and parkinsonism draw the most attention. More research with the dataset, combined with further developing the network architecture technique is needed to accurately map motor- and mental factors.

